# Identification and validation of a *Schistosoma japonicum* U6 promoter

**DOI:** 10.1186/s13071-017-2207-4

**Published:** 2017-06-05

**Authors:** Qing Li, Wan Wang, Nan Zhao, Pengcheng Li, Yue Xin, Wei Hu

**Affiliations:** 1 State Key Laboratory of Genetic Engineering, Ministry of Education Key Laboratory of Contemporary Anthropology, Department of Microbiology and Microbial Engineering, School of Life Science, Fudan University, Shanghai, 200433 China; 2 Key Laboratory of Parasite and Vector Biology of MOH, WHO Cooperation Center for Tropical Diseases, National Institute of Parasitic Diseases, Chinese Center for Diseases Control and Prevention, Shanghai, 200025 China

**Keywords:** *Schistosoma japonicum*, U6 promoter, sgRNA, RNAi, Lentivirus, Cas9 nuclease

## Abstract

**Background:**

RNA polymerase III promoters have been widely used to express short hairpin-RNA (shRNA), microRNA (miRNA), and small guide RNA (sgRNA) in gene functional analysis in a variety of organisms including *Schistosoma mansoni*. However, no endogenous RNA polymerase III promoters have been identified in *Schistosoma japonicum*. The lack of appropriate promoters in *S. japonicum* has hindered its gene functional analysis*.* Identification of functional promoters in *S. japonicum* is therefore in urgent need.

**Results:**

Via sequence alignment, a 347 bp sequence upstream from the coding region of *S. japonicum* U6 small nuclear RNA (snRNA) was identified, cloned, and named as *S. japonicum* U6 (sjU6) promoter. A sgRNA sequence named as sgRNA970 was designed, and its Cas9 nuclease guiding activity was confirmed by in vitro cleavage assay. The sjU6 promoter was ligated with sgRNA970 coding sequence by overlap PCR to generate a sjU6-sgRNA970 expression cassette. The expression cassette was inserted into a lentiviral plasmid to construct the pHBLV-sgRNA970 plasmid. First, we tested the sjU6 promoter activity in HEK293 cells by transfecting HEK293 cells with the pHBLV-sgRNA970 plasmid. RT-PCR amplification of the total RNA from the transfected HEK293 cells confirmed the presence of sgRNA970 transcript and indicated sjU6 promoter was functional to initiate transcription in HEK293 cells. Then we transduced the lentivirus expressing Cas9-ZsGreen fusion protein into 14 dpi schistosomula to test whether lentivirus was capable to induce exogenous gene expression in *S. japonicum*. Fluorescence microscopy and western blot results confirmed the expression of Cas9-ZsGreen fusion protein in *S. japonicum*. Therefore, this lentiviral system was adapted to test promoter activity in *S. japonicum*. Finally, we transduced 14 dpi *S. japonicum* with lentivirus produced from the pHBLV-sgRNA970 plasmid. RT-PCR amplification of the total RNA from transduced schistosomula confirmed the presence of sgRNA970 transcript and therefore indicated sjU6 promoter was functional to initiate transcription in *S. japonicum*.

**Conclusion:**

To our knowledge, sjU6 promoter would be the first identified and validated endogenous RNA polymerase III promoter in *S. japonicum*, which could be used for future CRISPR/Cas9 studies in *S. japonicum*.

**Electronic supplementary material:**

The online version of this article (doi:10.1186/s13071-017-2207-4) contains supplementary material, which is available to authorized users.

## Background

Genomic and transcriptomic data for the parasitic blood flukes *S. japonicum* and *S. mansoni* are now available [1–4]. In the post-genomic era, tools of functional genomics are essential to dissect gene functions and thus facilitating our understanding of their development and reproduction, survival in and interactions with the host, and pathogenicity. Combined with retrovirus mediated transgenesis, vector-based RNA interference (RNAi) has become a powerful tool for functional analysis of schistosome genes [5–7]. In recent studies, two types of promoters were used in RNAi studies of *S. mansoni*. The first type was the RNA polymerase II promoters which drove protein-coding mRNA transcription, including cytomegalomavirus (CMV) and *S. mansoni* actin (smActin) promoters. The smActin promoter was used to initiate the transcription of reporter protein mRNA (e.g. luciferase), as well as long fragment double-stranded RNA (dsRNA) [6, 7]. Although CMV promoter was primarily used to drive mRNA transcription, it was also used to initiate transcription of shRNA coding sequence tagged with a mCherry reporter [5, 8]. The second type were the RNA polymerase III promoters, mainly including the *S. mansoni* U6 (smU6) promoter. The smU6 promoter was identified to be active to initiate shRNA transcription in both human fibrosarcoma cells and schistosomula of *S. mansoni*, and therefore was widely used in schistosome RNAi studies [9, 10].

To date, no endogenous RNA polymerase III promoters in *S. japonicum* have been identified. The lack of available promoters in *S. japonicum* has hindered its gene functional analysis. Although CMV promoter was tested to be functional in *S. japonicum* [11], its larger size (600–700 bp compared to 250–350 bp of hU6 or smU6 promoter) narrows its applications in certain circumstances. For example, in a CRISPR/Cas9 system, a sgRNA expressing cassette, a Cas9 nuclease expressing cassette, as well as a donor template, are sometimes integrated in one vector to increase the transfection efficiency and homologous recombination [12]. Payload capacity of the vector is therefore an important consideration and the size of all expressing cassettes should be minimized, which means smaller promoters would be advantageous [13]. As CRISPR/Cas9 mediated genome editing is becoming increasingly important for gene functional analysis in a variety of species, identification of functional promoters for sgRNA and Cas9 nuclease expression is therefore in urgent need for *S. japonicum*.

In this study, we identified and cloned a 347 bp sequence upstream of the coding region of *S. japonicum* U6 snRNA. We named this 347 bp sequence as sjU6 promoter. We showed that this sjU6 promoter was functional to initiate transcription of a 98 nt sgRNA in HEK293 cells by plasmid transfection, as well as in 14 dpi schistosomula of *S. japonicum* by retroviral transduction. To the best of our knowledge, we identified and validated the first functional endogenous U6 promoter in *S. japonicum* for future genetic manipulation.

## Methods

### Parasites and cells

C-57 mice (18–20 g) were infected with ~100 *S. japonicum* cercariae percutaneously. At 14 days post-infection (dpi), mice were euthanatized and schistosomula were harvested. Eight to 10 pairs of schistosomula were cultured in 2 ml of RPMI 1640 medium with 10% FBS per well in a 12-well plate at 37 °C and 5% CO_2_.

HEK293 cell line, received from Professor Feng Qian (Fudan University) as a gift, was cultured in 2 ml of DEME medium with 10% FBS per well in a 6-well plate at 37 °C and 5% CO_2_.

### Prediction and isolation of a U6 gene promoter-like sequence

The 107 nt human U6 (hU6) snRNA (GenBank X59362.1), and the 109 nt *S. mansoni* U6 snRNA (GenBank L25920), were used as the queries to search for homologs in *S. japonicum* genomic database [3]. A 95.15% identical match was found and was named as sjU6 snRNA. A 379 bp sequence upstream of the sjU6 mRNA coding region was amplified by PCR using the primers sjU6P-F (5′-TTT ACA CGA CGT ATC AGT TAG TT-3′) and sjU6P-R(5′-AAT TTC GGC GGA TCA TTA TTA CA-3′), cloned into a PGEM-T-easy plasmid (Promega, Madison, USA) and sequenced. We named this PCR amplified sequence as sjU6 promoter and its regulatory activity was to be tested in HEK293 cells and *S. japonicum*.

### In vitro validation of sgRNA target site

To test whether our designed sRgNA could guide the cleavage of DNA of a genuine *S. japonicum* gene at the target site, we first performed an in vitro cleavage assay using a Guide-it^TM^ sgRNA In Vitro Transcription and Screening Systems (Takara, Shiga, Japan, Cat. No. 631439) as described in the users’ manual. In brief, a 20 bp sgRNA targeting sequence (5′-AAA TGA TGT CAC CTA GAA GA-3′), located 970 bp downstream from the transcription start site of the *S. japonicum* rhodopsin-like G-protein-coupled receptor (RL-GPCR) gene (Additional file 1: Dataset S1), was selected as the target site to synthesize a sgRNA (named as sgRNA970). The RL-GPCR gene contained only one exon but not any intron in *S. japonicum* genome therefore allowed direct amplification of the full coding sequence from the genomic DNA (gDNA) template. The 289 bp to 2306 bp region downstream from the RL-GPCR transcription start site, which contained the target site for sgRNA970, was PCR amplified from the *S. japonicum* genome as a target template for cleavage assay. sgRNA970, together with Cas9 nuclease supplied with the kit, was setup in a reaction to cleave the target template. The efficiency of cleavage was measured using 1% gel electrophoresis. Target template without any treatment, and target template incubate with Cas9 nuclease and sgRNA with a scrambled sgRNA targeting sequence (5′-GCA AAG GTC ATA AAG TCT AA-3′) served as negative controls.

### Construction of sgRNA expression vector

**Fig. 1:**
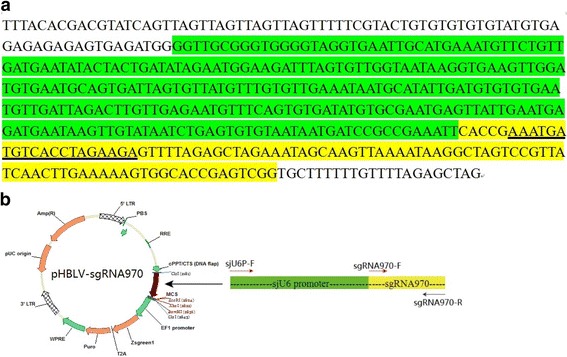
Schematic illustration of the sjU6-sgRNA970 expression cassette. **a** sjU6 promoter (*green*) was ligated with a 98 bp sgRNA coding sequence (*yellow*). The 20 bp sgRNA target sequence is underlineded in *black*. **b** The sjU6-sgRNA970 expression cassette was inserted into the pHBLV-CMVIE-ZsGreen-Puro lentiviral plasmid to construct the pHBLV -sgRNA970 plasmid

After being validated to be an effective target site for cleavage, the 20 bp PAM sequence for sgRNA970 was ligated into a linearlized CRISPR/Cas9 plasmid (pHBcas9/sgRNA Easy KO reagent, HanBio Ltd, Shanghai, China) to generate a 107 bp poly-T terminated sgRNA coding sequence for sgRNA970 as described in users’ manual. The sgRNA970 coding sequence was cloned from the CRISPR/Cas9 plasmid, and merged with sjU6 promoter, to generate a 468 bp sjU6-sgRNA970 expression cassette by overlap PCR (Fig. 1a). The sjU6-sgRNA970 expression cassette was inserted into the pHBLV-CMVIE-ZsGreen-Puro lentiviral plasmid at the ClaI sites between the 5′LTR and 3′LTR, to construct a lentiviral expression vector for sjU6-sgRNA970 (named as pHBLV-sgRNA970 plasmid) (Fig. 1b).

### Promoter activity assay in HEK293 cells

#### Transfection of HEK293 cells

To test whether the putative sjU6 promoter was functional in HEK293 cell line, 2 μg of pHBLV-sgRNA970 plasmid and 4 μg of Polyethyleneimine (Polysciences, Warrington, USA, Cat. No. 23966) per well in 12-well plates were used to transfect HEK293 cells. Forty eight hour after transfection HEK293 cells were collected by a 2 min centrifugation to remove the supernatant. Recovered samples were washed twice with 1.5 ml of 1× PBS, to remove remaining plasmid in the tube.

#### Total RNA extraction and cDNA synthesis

Total RNA was isolated using TriZol reagent as previously described [14]. Total RNA was treated with DNAseI to eliminate DNA contamination. Total RNA was quantified on a Nanodrop 2000 spectrophotometer (Thermo Scientific, Waltham, MA) and the quality was measured by the A260/A280 ratio. Five hundred nanogram of DNAseI-treated total RNA was used to synthesize total cDNA with random primers using a PrimeScript RT Master Mix kit (Takara, Cat. No. RP036A) in a total volume of 15 μl.

#### sgRNA transcript detection

**Fig. 2:**
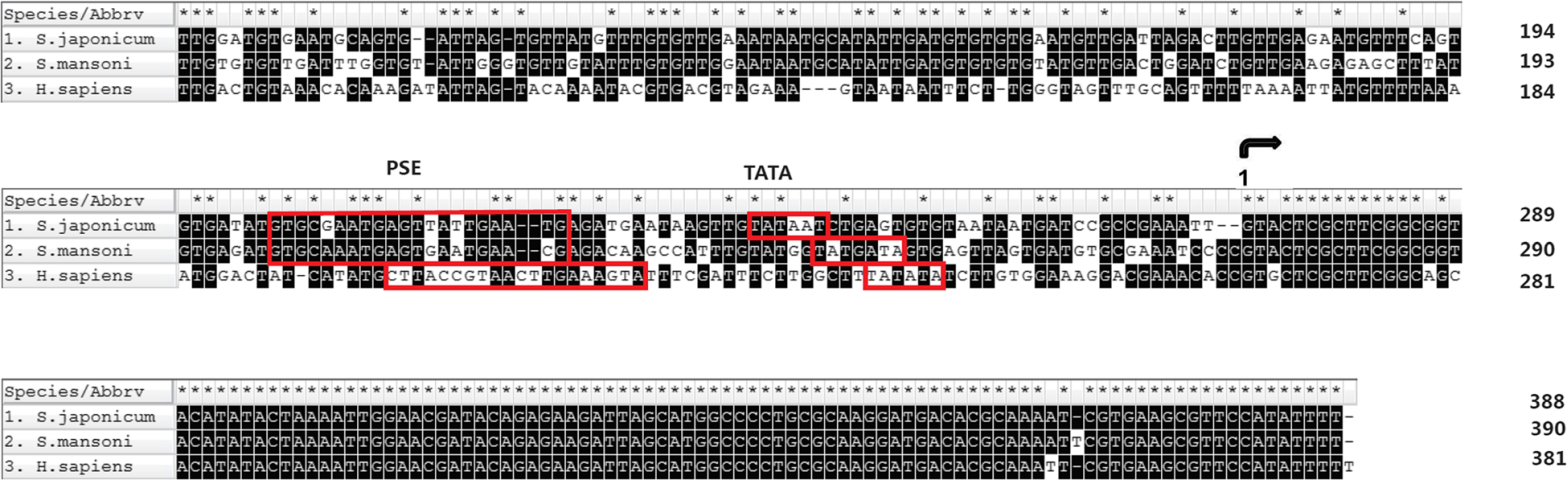
Multiple sequence alignment of the U6 genes of *Schistosoma japonicum*, *S. mansoni* and *Homo sapiens*. The multiple alignment was performed with Mega 7, and the following sequences were aligned: U6 snRNA of *S. japonicum*, *S. mansoni* (GenBank L25920) and *H. sapiens* (GenBank X59362.1); U6 promoter regions of *S. japonicum*, *S. mansoni* (GenBank HQ540317), and *H. sapiens* (GenBank X07425). Residue 1 (curved *black* arrow is the first nucleotide of U6 snRNA. Promoter elements including OCT, PSE, and TATA box are indicated in *red* colored rectangles

To detect the expression of the 98 bp sgRNA970 transcript initiated by the putative sjU6 promoter, PCR was performed using the primers sgRNA970-F (5′-CAC CGA AAT GAT GTC ACC TAG A-3′) and sgRNA970-R (5′-CCG ACT CGG TGC CAC TTT TTC A-3′) to amplify the 98 bp sgRNA cDNA fragment from pHBLV-sgRNA970 plasmid transfected HEK293 cell cDNA sample (Fig. 2b). HEK293 cells without transfection served as a blank control. RNA template from transfected 293 cells without reverse transcription served as a negative control.

### Lentivirus transduction assay in *S. japonicum*

#### Lentivirus transduction of S. japonicum

To test whether lentiviral system could be used for gene delivery in *S. japonicum*, pHBLV-CMVIE-Cas9-ZsGreen lentivirus, which contained a coding sequence of Cas9-ZsGreen fusion protein regulated by a CMVIE promoter, was purchased from HanBio Ltd (Shanghai, China). The virus was diluted into 10^7^ CFU/ml in 1640 medium with 10% FBS and used to transduce cultured 14 dpi schistosomula; 8 μg/ml of polybrene was added to each well in 12-well plates to increase transduction efficiency. Forty eight hour after viral exposure, schistosomula were cultured in fresh RPMI 1640 medium with 10% FBS for additional 48 h before to be harvested. HEK293 cells transduced with pHBLV- CMVIE-Cas9-ZsGreen lentivirus served as a positive quality control.

#### Fluorescence reporter detection

To detect the expression of Cas9-ZsGreen fusion protein, living schistosomula and HEK293 cells transduced by pHBLV-CMVIE-Cas9-ZsGreen lentivirus were placed in PBS and observed under a fluorescence microscope (Zesis) for ZsGreen signal. Schistosomula without transduction served as a blank control.

#### Western blot detection of Cas9 nuclease

After microscopic observation, schistosomula and HEK293 cell samples were lysed for Western Blot detection of Cas9 expression as described [12]. Briefly, Cas9 expression was detected by a rabbit monoclonal Anti-Cas9 primary antibody (Cat. No. ab210752; 1:5,000; Abcam, Cambridge, UK) and a goat anti-rabbit polyclonal IgG-HRP secondary antibody (Cat. No. ab6721; 1:5,000; Abcam). As a quality control, tubulin expression in both schistosomula and HEK293 cell samples were detected by a mouse monoclonal anti-tubulin primary antibody (Cat. No. ab44928; 1:5000; Abcam) and a goat anti-mouse polyclonal IgG-HRP secondary antibody (Cat. No. ab6789; 1:5000; Abcam). Schistosomula samples without transduction served as a blank control. To rule out the possibility that pHBLV- CMVIE-Cas9-ZsGreen lentivirus contained Cas9 protein leading to subsequent false positive result, 20 μl of lentivirus were lysed and sampled, to serve as another negative control.

### Promoter activity assay in *S. japonicum*

#### Lentivirus production

HEK293T cells were transfected with the pHBLV-sgRNA970 plasmid together with the pSPAX2 and the pMD2G plasmid, and aided with the Lipofiter^TM^ transfection reagent (HanBio Ltd), to produce lentivirus (named as pHBLV-sgRNA970 lentivirus) by HanBio Ltd. Transfected HEK293T cells were cultured in 10% FBS in DMEM, supplemented with 100 U of penicillin and streptomycin, at 37 °C under 5% CO_2_ in air. Culture media were replaced after 16 h to remove transfection reagent and residual plasmid. After a further 48 h supernatant containing virus were harvested and filtered through 0.45 μM pore size membrane to remove cell debris. Viral supernatant was incubated with 2U/ml of DNAseI gDNA (Cat. No. AM1907 Invitrogen, Waltham, USA). Subsequently, viral supernatant was concentrated by centrifugation at 4 °C, 72,000× *g* for 120 min. The pellet of concentrated virions was resuspended in DMEM. Viral titer was determined by cell assay as described [6].

#### Transduction of schistosomula

To test whether the putative sjU6 promoter was functional in *S. japonicum*, the pHBLV-sgRNA970 lentivirus was used to transduce cultured 14 dpi schistosomula as mentioned before. Schistosomula without transduction served as a blank control.

#### Virus entrance analysis

Schistosomula gDNA samples were extracted using a Qiagen DNeasy Blood & Tissue Kit (Cat. No. 69504, Qiagen, Hilden, Germany). Entrance of the lentivirus into schistosomula cells after transduction was detected by PCR amplification of the sjU6-sgRNA970 expression cassette using transduced schistosomula gDNA templates, and using the primers sjU6P-F and sgRNA970-R. PCR using gDNA templates from schistosomula without transduction served as blank controls.

#### sgRNA transcript detetion

Schistosomula total RNA was extracted and cDNA was synthesized as mentioned before for HEK293 cells. The quality of schistosomula cDNA was tested by PCR amplification of the internal control gene PSMD (26S proteasome non-ATPase) using the primers PSMD-F (5′-CCT CAC CAA CAA TTT CCA CAT CT-3′) and PSMD-R (5′-GAT CAC TTA TAG CCT TGC-3′) [15].

To detect the expression of the 98 bp sgRNA970 transcript initiated by the putative sjU6 promoter, PCR was performed using primer sgRNA970-F and sgRNA970-R to amplify the 98 bp sgRNA cDNA fragment from pHBLV-sgRNA970 lentivirus transduced schistosomula cDNA sample. PCR using cDNA templates from schistosomula without transduction served as a blank control. PCR using RNA templates from transduced schistosomula without reverse transcription served as a negative control. PCR amplification of the 445 bp fragment of the sjU6-sgRNA970 expression cassette from schistosomula cDNA was performed using primer sjU6P-F and sgRNA970-R to also serve as a negative control.

## Results

### Promoter region of the sjU6 snRNA

A 95% identical match to the smU6 snRNA was found in *S. japonicum* genomic database. A 347 bp sequence upstream of the sjU6 mRNA coding region was amplified by PCR, cloned into a PGEM-T-easy plasmid. The T-clone sequence was in general identical to that in database, however with a few deletions at the 5′ end. Therefore, the actual PCR amplicon was only 347 bp in length. We named this 347 bp sequence as sjU6 promoter.

The sjU6 gene was aligned with *H. sapiens* and *S. mansoni* U6 genes (Fig. 2). The sjU6 snRNA sequence is 95.15% identical to its human and *S. mansoni* orthologues. While the sequence of the putative sjU6 promoter is 49.86% identical to the smU6 promoter, it is only 36.57% identical to the hU6 promoter. Alignment of sjU6 promoter and smU6 promoter also showed that most matches were found within the 280 bp region upstream from the 3′ end of the promoters, indicating that the core regulatory elements of the sjU6 promoter were located within the 273 bp region upstream of the sjU6 snRNA coding region. We attempted to identify and predict some promoter elements within this 273 bp region based on more or lesser sequence identity to the human and *S. mansoni* promoter regulatory motifs; octamer motif (OCT), 253-AAT TGC AT-246; proximal sequence element(PSE), 71-GTG CGA ATG AGT TAT TGA ATG-51; TATA box, 36-TATAAT-31;

### sgRNA970 is effective to guide DNA cleavage

**Fig. 3:**
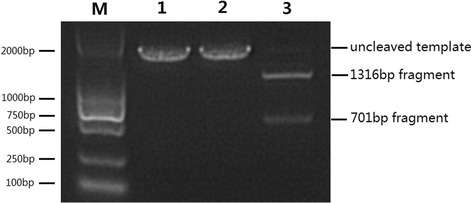
Analysis of cleavage products. The sgRNA970 was tested against the target DNA template derived from the *S. japonicum* RL-GPCR gene. Lane M: DL2000 DNA marker; Lane1: untreated template; Lane 2: template treated with Cas9 nuclease and scrambled sgRNA; Lane 3: template treated with Cas9 nuclease and sgRNA970

To test whether sgRNA970 could guide the Cas9 nuclease to cleave the DNA template derived from the *S. japonicum* RL-GPCR gene at the target site, we first performed an in vitro cleavage assay. Gel electrophoresis showed that the 2017 bp target template was almost completely cleaved into two expected fragments of unequal size (701 bp and 1,316 bp, respectively), indicating that Cas9 nuclease was guided by sgRNA970 to cleave the template at the target site (Fig. 3).

### sjU6 promoter is active in HEK293 cells

**Fig. 4:**
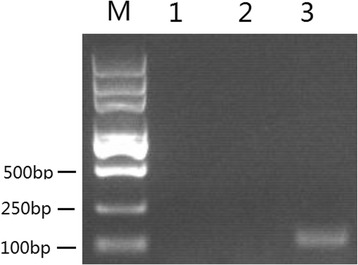
sjU6 promoter is active in HEK293 cells. HEK293 cells were transfected with 2ug of pHBLV-sgRNA970 plasmid and the transcript of the 98 bp sgRNA970 was detected by RT-PCR after 48 h. Lane M: Trans2K DNA maker; Lane 1: unstransfected HEK293 cell cDNA; Lane 2: transfected HEK293 cell RNA treated with DNAseI, but without reverse transcription; Lane 3: cDNA synthesized from transfected HEK293 cell RNA treated with DNAseI

To test whether the putative sjU6 promoter was capable to drive the transcription of sgRNA970, pHBLV -sgRNA970 plasmid was used to transfect HEK293 cells. The presence of the 98 nt sgRNA970 transcript was analyzed by PCR from transfected HEK293 cell cDNA sample. Gel electrophoresis result showed that a 98 bp product was observed from transfected 293 cell cDNA samples after PCR amplification, confirming the presence of the sgRNA970 transcript (Fig. 4). To rule out the possibility such amplification was resulted from residual pHBLV-sgRNA970 plasmid contamination, PCR amplification of total RNA treated with DNAseI was used as a negative control, and no product was observed. Taken together, our results showed that the 347 bp putative sjU6 promoter was functional to initiate the transcription of a 98 nt sgRNA in 293 cells.

**Fig. 5:**
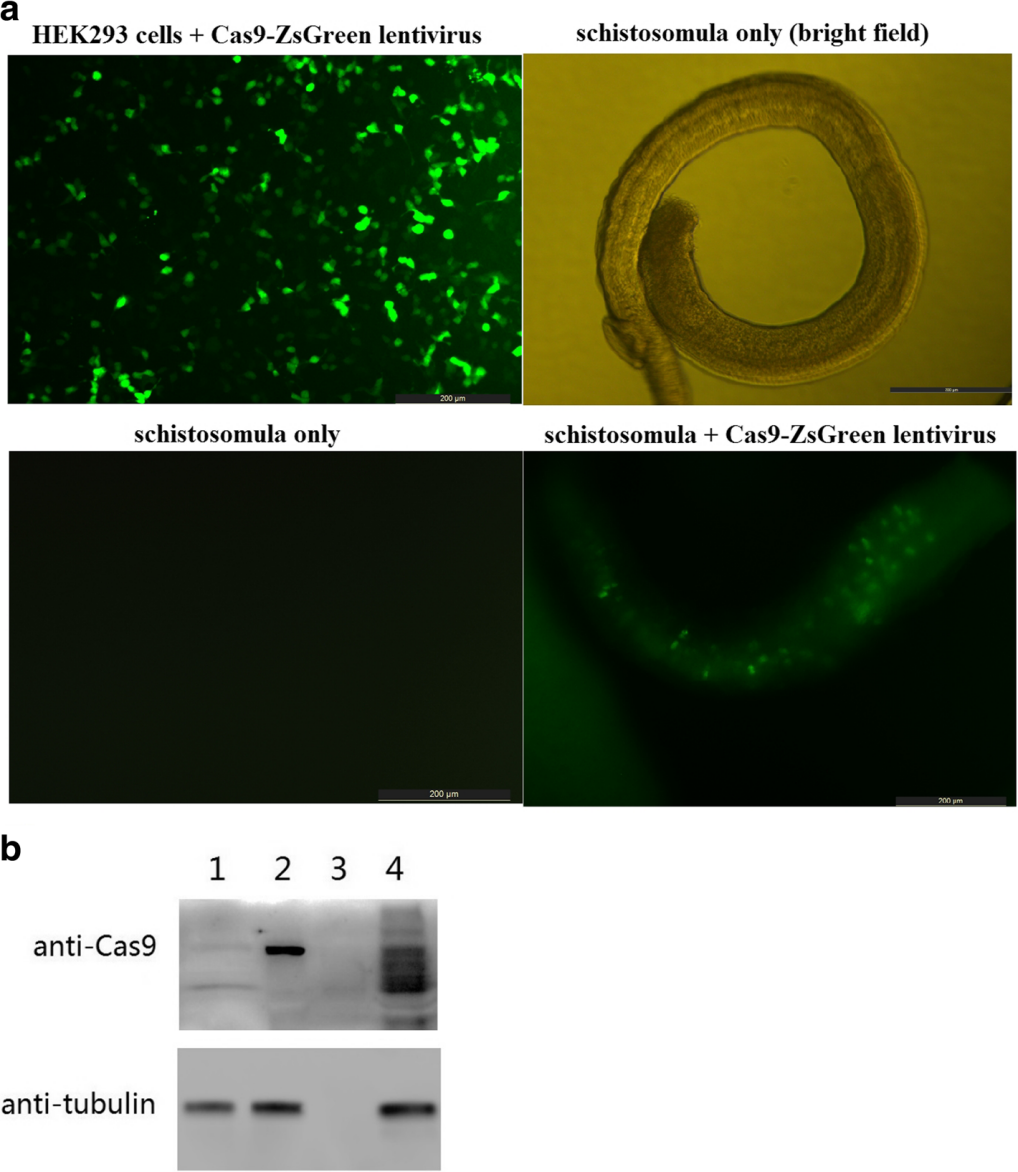
Lentivrius induced Cas9-ZsGreen expression in *S. japonicum*. Schistosomula were transduced with pHBLV- CMVIE-Cas9-ZsGreen lentivirus. **a** Fluorescence microscopic detection of the ZsGreen fluorescent signal in lentivirus-transduced schistosomula. HEK293 cells transduced with lentivirus served as a positive control. Schistosomula without lentivirus transduction served as a negative control. **b** Western blot detection of Cas9-ZsGreen fusion protein expression. Lane 1: schistosomula without lentivirus transduction; Lane 2: HEK293 cells transduced with lentivirus as positive control; Lane 3: lysed virus as negative control; Lane 4: schistosomula transduced with lentivirus

### Lentivirus induced Cas9-ZsGreen expression in *S. japonicum*

To test whether the lentiviral system could be used for gene delivery in *S. japonicum*, pHBLV-CMVIE-Cas9-ZsGreen lentivirus was used to transduce schistosomula and HEK293 cells. Fluorescence microscopy showed that, similar to transduced HEK293 cells, schistosomula transduced by pHBLV-CMVIE-Cas9-ZsGreen lentivirus displayed green fluosrescence signal around the intestine, indicating the expression of Cas9-ZsGreen protein (Fig. 5a). However, to our surprise, no ZsGreen signal was detected in tegument which exposed directly to lentivirus. No ZsGreen signal was detected in schistosomula without transduction.

Western blot result also showed that, in transduced schistosomula sample, an approximately 191 KDa band, identical to the position of the band for Cas9-ZsGreen in transduced HEK293 cell sample, were observed, indicating the expression of Cas9-ZsGreen fusion protein (Fig. 5b). No similar bands were observed in blank schistosomula sample and lysed lentivirus sample.

Taken together, our results indicate that the lentiviral system was capable to deliver exogenous genes into 14 dpi *S. japonicum* and therefore could be used to test the promoter activity.

### sjU6 promoter is active in *S. japonicum*

**Fig. 6:**
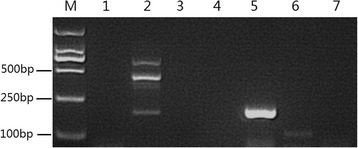
sjU6 promoter is active in *S. japonicum*. Schistosomula were transduced 14 dpi with pHBLV-sgRNA970 lentivirus for 48 h and cultured for additional 48 h after viral exposure. Lane M: DL2000 DNA marker; Lane 1: amplification of the 445 bp sjU6-sgRNA970 expression cassette from gDNA of untrasduced schistosomula; Lane 2: amplification of the 445 bp sjU6-sgRNA970 expression cassette from transduced schistosomula gDNA; Lane3: amplification of the 98 bp sgRNA970 from untransduced schistosomula cDNA; Lane4: amplification of the 98 bp sgRNA970 from transduced schistosomula RNA treated with DNAseI, but without reverse transcription; Lane 5: amplification of the PSMD internal control gene from cDNA synthesized from transduced schistosomula RNA treated with DNAseI; Lane 6, amplification of the 98 bp sgRNA970 from cDNA synthesized from transduced schistosomula RNA treated with DNAseI; Lane 7: amplification of the 445 bp sjU6-sgRNA970 expression cassette from cDNA synthesized from transduced schistosomula RNA treated with DNAseI

To test whether the putative sjU6 promoter was functional in *S. japonicum*, the pHBLV-sgRNA970 lentivirus was used to transduce 14 dpi schistosomula. PCR amplification of the 445 bp fragment of the sjU6-sgRNA970 expression cassette from transduced schistosomula gDNA indicated that the sjU6-sgRNA970 expression cassette carried by lentivirus had been delivered into the schistosomula cells (Fig. 6). The presence of the 98 nt sgRNA970 transcript was confirmed by PCR amplification from transduced schistosomula cDNA sample, however the band in agarose gel was significantly dimmer than that amplified from the transfected HEK293 cDNA sample (Fig. 4) and from pHBLV-sgRNA970 lentivirus transduced HEK293 cell cDNA sample (data not displayed). To rule out the possibility that such amplification was resulted from the sjU6-sgRNA970 expression cassette integrated in schistosomula gDNA, PCR amplification of total RNA treated with DNAseI was used as a negative control, and no product was observed. To rule out the possibility that such amplification was resulted from cDNA template reverse-transcribed from residual viral genomic RNA, PCR amplification of the 445 bp fragment of the sjU6-sgRNA970 expression cassette from schistosomula cDNA was also performed, and no product was observed. Taken together, our results indicate that facilitated by lentivirus, the sjU6-sgRNA970 expression cassette had been integrated into the schistosomula gDNA, and subsequently, the sgRNA transcription was initiated by the active sjU6 promoter. We therefore confirmed that sjU6 promoter was active in *S. japonicum*.

## Discussion

Here we identified and validated a sjU6 promoter active in both HEK293 cells and *S. japonicum*. Previous studies showed that the sequence of U6 snRNA were highly conserved across species, however the promoter region of the U6 gene diverged substantially in different species [9, 10]. Thus prediction of the sjU6 promoter based on sequence alignment of the promoter region with U6 promoters in other species would be difficult and few softwares for prediction are now available. However, it is known that most U6 promoters are located within the 500 bp region upstream of U6 snRNA coding region. Therefore in previous studies, putative *S. mansoni* U6 promoter and *Plasmodium yoelii* U6 (pyU6) promoter was identified simply by cloning the 250–350 bp upstream sequence of the U6 snRNA coding region [10, 12]. Consistent with previous studies, our result showed that the active sjU6 promoter was also located within the 350 bp region upstream of U6 snRNA coding region.

In previous studies, two different methods were used for promoter activity assay. The first method, as used in the validation of smU6 promoter and porcine 7SK promoter activity, linked a shRNA to the putative promoter in a plasmid [10, 16]. The shRNA plasmid was co-transfected with a reporter (e.g luciferase or GFP) plasmid into target organisms. If the reporter was significantly knocked down by the shRNA, the putative promoter would be considered as functional. However, knockdown assay only provided indirect evidence for the promoter activity. The second method, as used in this study and the validation of pyU6 promoter activity, provided a direct proof: a sgRNA coding sequence was linked to the putative promoter in a vector to transfect the target organism, and if the sgRNA transcript was detected by PCR amplification of the cDNA, activity of the putative promoter could be confirmed [12]. If appropriate controls were given, this method circumvented the uncertainty of statistics and therefore provided directly evidence of the promoter’s activity.

Based on the method used in pyU6 promoter activity assay, we modified the method for gene delivery into *S. japonicum*. As HEK293 cells were very easy to be transfected, plasmid and lentivirus were used as positive controls of delivering the sjU6-sgRNA970 expression cassette into schistosomula. Our results showed this sjU6 promoter was functional to initiate transcription of a 98 nt sgRNA in HEK293cells by plasmid transfection and lentiviral transduction, as well as in 14 dpi *S. japonicum* by lentiviral transduction. However, agarose gel electrophoresis results showed that the band representing the 98 bp sgRNA product amplified from transduced schistosomula cDNA sample was significantly dimmer than that from the transfected HEK293 cell cDNA sample, indicating that the sgRNA was less abundant in schistosomula than in HEK293 cells. This was consistent with the result that in schistosomula transduced by pHBLV-CMVIE-Cas9-ZsGreen lentivirus, both the ZsGreen fluorescence signal and the Cas9 Western blot signal were significantly weaker than those in HEK293 cells. We inferred that the larger body size of 14 dpi schistosomula had a smaller surface area/volume ratio, therefore reduced transduction efficiency and exougenous gene expression. Furthermore, although a relatively high concentration (10^7^ CFU/ml) of lentivirus was used to transduce schistosomula, ZsGreen fluorescence signal was only detected around the schistosomula intestine, but not in the tegument which exposed directly to lentivirus. This suggested that the tegument might possess special surface structures and confer *S. japonicum* resistance against viral invasion, and therefore significantly reduced the transduction efficiency. However, our result provided evidence that lentivirus could be used to transduce 14 dpi *S. japonicum*, which could be potentially valuable when gene functional analysis must be performed at larger worm stages.

Previous studies showed that green fluorescence protein (GFP) could be expressed under the regulation of a CMV promoter in *S. japonicum* [11]. In this study, Cas9-ZsGreen-expressing lentivirus was also used to transduce 14 dpi *S. japonicum*. Fluorescence microscopy and western blot results showed that the Cas9-ZsGreen fusion protein was expressed in schistosomula. Our results not only confirmed that lentiviral system could be used for gene delivery in *S. japonicum*, but also indicated that the we were able to express Cas9 nuclease in *S. japonicum* and its expression could be monitored directly by the visible ZsGreen reporter. Therefore, the value of this functional sjU6 promoter not only manifests in its application in vector-based RNAi studies, but more importantly, in the future application of CRISPR/Cas9 mediated genome editing. In future, by integrating a CMV-Cas9 and a sjU6-sgRNA expression cassette in a lentiviral vector, we would attempt to simultaneously express a Cas9 nuclease and a sgRNA for precise genome editing in *S. japonicum*. The relatively low lentivirus transduction efficiency in 14 dpi schistosomula would limit the amount of expressed Cas9 nuclease and sgRNA, as well as reduce the efficiency of genome editing. Therefore, only a small percentage of schistosomula cells might be edited and most cells would remain wild-type. In this case, next generation sequencing should be used to sequence every DNA molecule in the sample so that the rare mutation could be detected. Previous studies also showed that germline transgenesis could be achieved in *S. masoni* by retrovirus transduction of eggs [6, 17]. Therefore, transduction of eggs might be a better approach to generate cercariae that carry the Cas9 and sgRNA expression cassette in every cell, hence allowing an entire cercaria to be edited, so that a heritable strain with precise gene modification can be generated.

In this preliminary study, only the 350 bp upstream sequence of the U6 snRNA coding region was tested for its promoter activity. However, upstream sequences of different lengths should also be tested in future studies to determine the minimum length of sequence containing core promoter elements, and the optimal length of sequence with maximum transcription activity.

## Conclusions

To the best of our knowledge, in this study we identified and validated the first functional endogenous sjU6 promoter in *S. japonicum*. We showed that sjU6 promoter was capable to initiate the transcription of sgRNA970, and that sgRNA970 was effective to guide Cas9 nuclease to cleave the target DNA template derived from *S. japonicum* RL-GPCR gene. We also showed that the Cas9-ZsGreen fusion protein could be expressed in *S. japonicum* using a lentiviral vector. Therefore, the identification of the sjU6 promoter not only allowed us to perform vector-based RNAi, but also laid a sound foundation for future CRISPR/Cas9 mediated genome editing in *S. japonicum*.

## Additional file

Additional file 1: Dataset S1. Genomic sequence of the *S. japonicum* rhodopsin-like G protein coupled receptor gene. The1,023 bp coding sequence of the RL-GPCR gene is highlighted in *red*; the 20 bp PAM sequence for sgRNA970 is underlined in *black*; the forward primer for amplification of the 2,017 bp target template in in vitro cleavage assay is underlined in *blue*; the reverse primer for amplification of the 2017 bp target template in in vitro cleavage assay is underlined in *green*. (PDF 8 kb)

## Abbreviations

CMV: Cytomegalomavirus
CRISPR: Clustered Regularly Interspaced Short Palindromic Repeats
dpi: Days post-infection
dsRNA: Double-stranded RNA
gDNA: Genomic DNA
GFP: Green Fluorescence Protein
miRNA: microRNA
pyU6 promoter: *Plasmodium yoelii* U6 promoter
RL-GPCR: Rhodopsin Like G-Protein-Coupled Receptor
RNAi: RNA interference
sgRNA: small guide RNA
shRNA: Short hairpin RNA
sjU6 promoter: *Schistosoma japonicum* U6 promoter
smActin promoter: *Schistosoma mansoni* Actin promoter
snRNA: small nuclear RNA
