# Supplementary material for: Identification and validation of a Schistosoma japonicum U6 promoter
Source: Parasit Vectors. 2017 Jun 5;10:281. doi: 10.1186/s13071-017-2207-4 (PMC5460494; doi:10.1186/s13071-017-2207-4)
Supplement: Additional file 1: Dataset S1. — Genomic sequence of the S. japonicum rhodopsin-like G protein coupled receptor gene. The1,023 bp coding sequence of the RL-GPCR gene is highlighted in red; the 20 bp PAM sequence for sgRNA970 is underlined in black; the forward primer for amplification of the 2,017 bp target template in in vitro cleavage assay is underlined in blue; the reverse primer for amplification of the 2017 bp target template in in vitro cleavage assay is underlined in green. (PDF 8 kb) [file 13071_2017_2207_MOESM1_ESM.pdf]

S1. Genomic Sequence of the *S.japonicum* Rhodopsin-Like G protein Coupled Receptor Gene. The1,023 bp coding sequence of the RL-GPCR gene is highlighted in *red*; the 20 bp PAM sequence for sgRNA970 is underlined in *black*; the forward primer for amplification of the 2,017 bp target template in *in vitro* cleavage assay is underlined in *blue*; the reverse primer for amplification of the 2,017 bp target template in *in vitro* cleavage assay is underlined in *green*.

ATATATATATAGTAACATTTGACATGATTTTCTAATACTCTGATTATACATTGAATTATGTA
ATAAAGATTTAATATGTAATAATATTAATATAATTCTATTATTTCATTAAAGAATAATTTTTT
TAAAAAGAAAGAGAGAGAGAGAGAATAAAAAAGATTATCATAATGGAACAATATAATA
AAACAACAGATTATAGTCATTTAGTTGGAAGTTTTCGTGCATATATTTCACCAATTATTG
CTATAATTGGTATACTAGGTAATTGTTTTATTATTATTATATTTCTACAAGAGAAAATTAAA
TCAAGATTTTCAATTTATTCAATATTTTTAGCTATTGCTAATATTATCATATTAATAATGAAT
ACATTAATTGATGATTTTCTTGGACGTGGTTTATATTATGCAACTTATCAACAAATCTATT
TTAAATTAGATACAATATCAAAATTTTGGTGTAAATTTGTTGAATATCTATCGAATACAAT
GTATTTTACATCATCATATTTAATTGTTATCTTTTCAATTGATCGTTTATTAACTATTCATCA
ACCGATTACATTCTATTCAATATATCATAAACAATGGGCATTAATTGCATGCATTATTATAT
ATTTACTTGGTATAATTAGTAATTCACCATTATTATTTGTACAAACATTAATGATAGATAAA
TCAAGTAGAACAAATTTTACATGTAGAATGATATCAGAACATCCAATTGCAAAATTTAC
AATAACATTTGAAACAATCGCTACATTTACATTACCATTTTGTCTTGTTTTAATATTAAAT
ATTTTCATTTGTATACAATTATGGACATTAAAAATGAATAGAATAGCATTATTACCAACTG
ATTCATCAAGGAATAGTATGGAGATGGGAAGAGTATTTGGACATTTAGCTTTAAGTACT
GTATTCTTATTATTGTATTTACCAATGGTATGTTTTGTATTAATACGTTTAAGTCAAACTTT
GTTACATGTTGATAGGCATAAACTGTATGCAATGCGTATTATTGACTTATCTAGATTATTC
TCATCTGTTAAAGATATTACTTATGGAGTTAACTTTTTCCTGTATTTAATATTCTTGAAAA
ATTTTAGACAACGATTTAATCATTTGATATGTTGTAAAATGATGTCACCTAGAAGACGGA
CAACATTGCATAGACCACATGAGAGATGAATGATTATGTAATACATTATTACTTTATGTG
GCTTGATTAACAGACTTCTGTTAGCATTGTTTTTTCTCTTTTTGCAGTTAATAGTTTTTTG
CATCAAAAATACACATCTTGTTAATAGATTCACTTGATATTGATTTTTTTTGTTAAAAAGC
GCTTTACTCGATATAAGAACTAAATCTAGTGAATAAAATTCATTTATTCTATTTAGTTTAC
TAACAAATGATTTTAGTAGTTACATCATTTTAATTCAAATTATGAAAAATCTACCCACGAT
TACGAGTTAATTGTTAGAATTAGTCTATTTGACGAGATATACAAAAGATGCTACCGGTGA
ACTCTTATTAGATTTGAAGTGATTGCCTTACCGACATGCACAAAAAATTACTCAGTGTC
ACAGTTGTTTGTGTTCCTAAATCGTTAAATATTATGATGTCATCCAAATTGATAGAAATT
GATGAAAGGATAAAATTAGAAGACAGTATCATGCTGCTTGATATTTCCATTAATACAGA
GATAGGATACATCTTAAATAATGGAACGACTTTTGAGTCATAACATATAAGTTGAATCAT
CCTTGTCCAGAAATTTCGTAGAATAACCCTACAGTATAGCTATAAGACAGATACCGAAG
TGAAACATAAGATTTAGGATTGTTCATGCAACAGTAGAGGTTATCCGTAATATTTTCAAT
GGTTACAAGTAAAATGCATCACGCGTATTTGGTCATTTTACTGCTTTTTTATTGAATGAA
TACTAAATATTAGTGAATTTTCAAGGTATTTGCTATGCAGGTCTGTACTAAAGTAACAAG
CTATAGTCTTAGAAAAGAAACATAGATATCCACTGGTTATTTATTTTATGAAAATTATAGC
CATTTCTGATAAGGAACTTAGTAACAAAATATTGATTGTGGGTTAGTTGGATTACAGTTA
AATTAGCTTGTCATGTCAACAACTTATAAGGAAATTAAGTACAACTTGTATATAATTTGT
CGCATCTTTTTATAAAAAGTTAGCTGACATTACGAGATGAAATTTCTCCTCAATATATCTA

GTCCCGGTTCCGAAGAAATGTTTTAACTATCTCGTTGATTCATTGCTGAGTAGTGACTAA
TGTAATGTCTTTTTAACTCATAGTACTGATCGAATTCTATCAAAGATTACGCGAATAGTT
CAAGTGGCTCAACACTGTGCAGACTTTTTCGATGTAAGCTGGTTGAATGAAGTTGGCA
GGAAGTATTTGGTCACCTCAACTATAAACTCGACCTAATATATCGGTTTGGTCTATAAAG
ACCCCAACTGTTCTTTAAAGAACATAACACCATGATAAACTTTCTAACTAGCTAATAAA
